# Supplementary figures and images for: Projection-based stereolithography for direct 3D printing of heterogeneous ultrasound phantoms
Source: PLoS One. 2021 Dec 9;16(12):e0260737. doi: 10.1371/journal.pone.0260737 (PMC8659365; doi:10.1371/journal.pone.0260737)

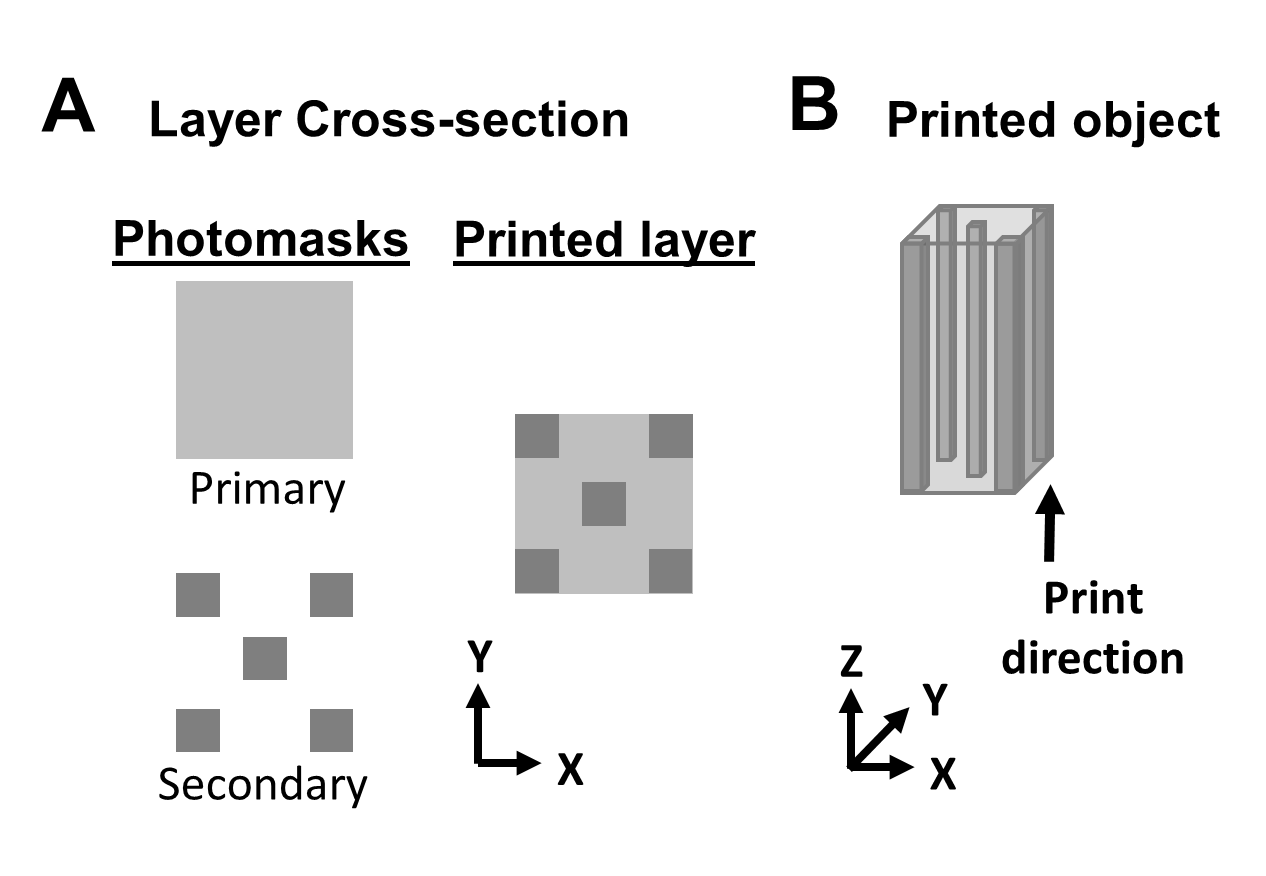

Supplement: S1 Fig — (A) A Python script adds regions of secondary exposure to the primary background exposure to produce the illumination pattern for the printed object at each slice. (B) This process results in a final monolithic gel with different levels of photocuring. (TIF) [file pone.0260737.s001.tif]

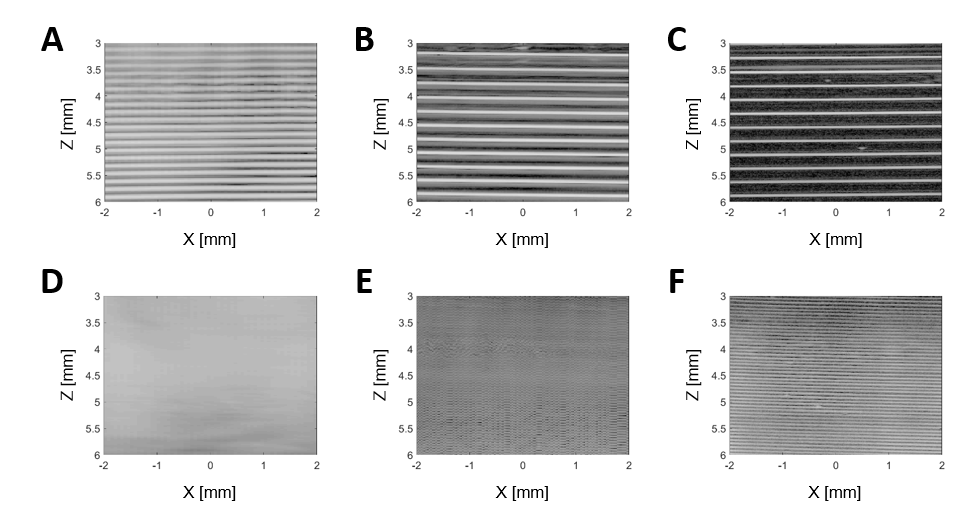

Supplement: S2 Fig — B-mode images at (A) 12 MHz, (B) 30 MHz, & (C) 50 MHz of Phantom 1 with 200-μm layers showing the hyperechoic signal presenting at layer interfaces. B-mode images at (D) 12 MHz, (E) 30 MHz, & (F) 50 MHz of 50-μm layer Phantom 2. (TIF) [file pone.0260737.s002.tif]

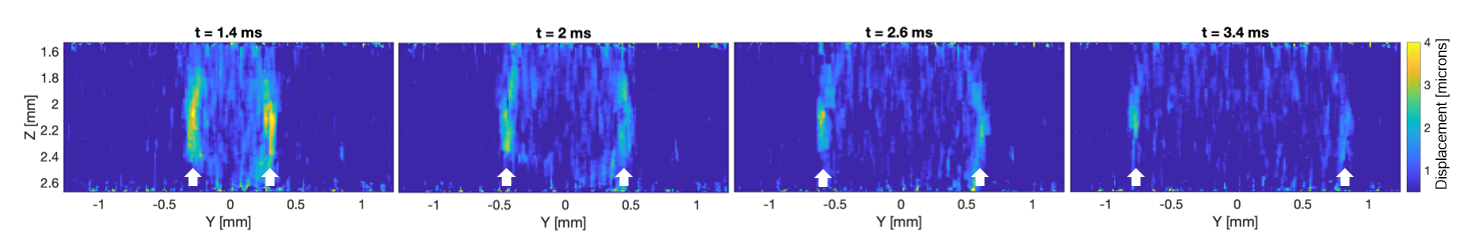

Supplement: S3 Fig — Images of US-based axial displacement estimates showing shear wave propagation (wave fronts identified with white arrows) at four time-points following an acoustic radiation force impulse in an anisotropic elasticity phantom (0° in Phantom 14). (TIF) [file pone.0260737.s003.tif]

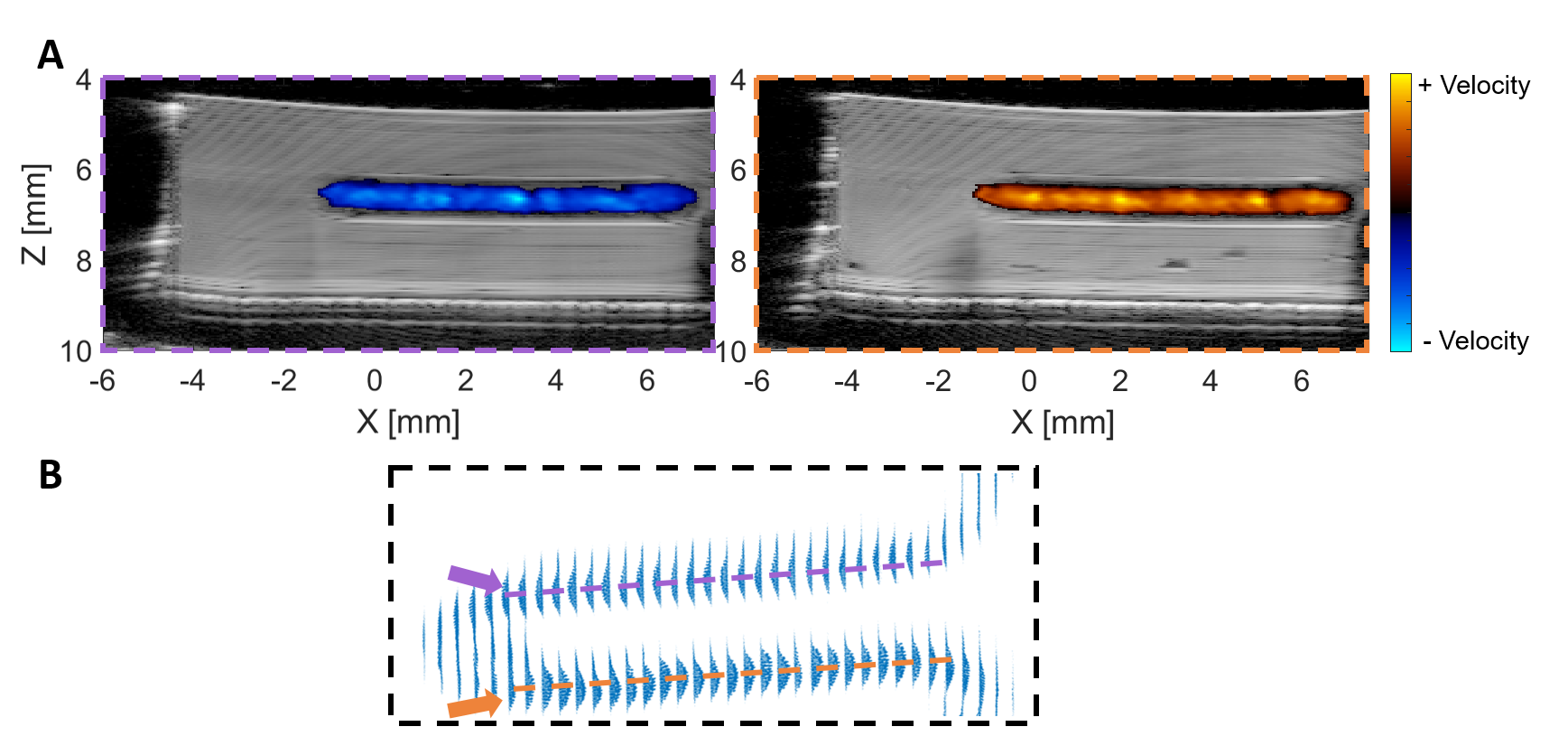

Supplement: S4 Fig — (A) Doppler data overlaid on B-mode images and (B) cut-out of the flow-velocity vector data from Fig 5C denoting the two imaging planes (distinguished by orange or purple dashed lines/arrows) shown through channels in Phantom 16 with opposite flow directions. (TIF) [file pone.0260737.s004.tif]

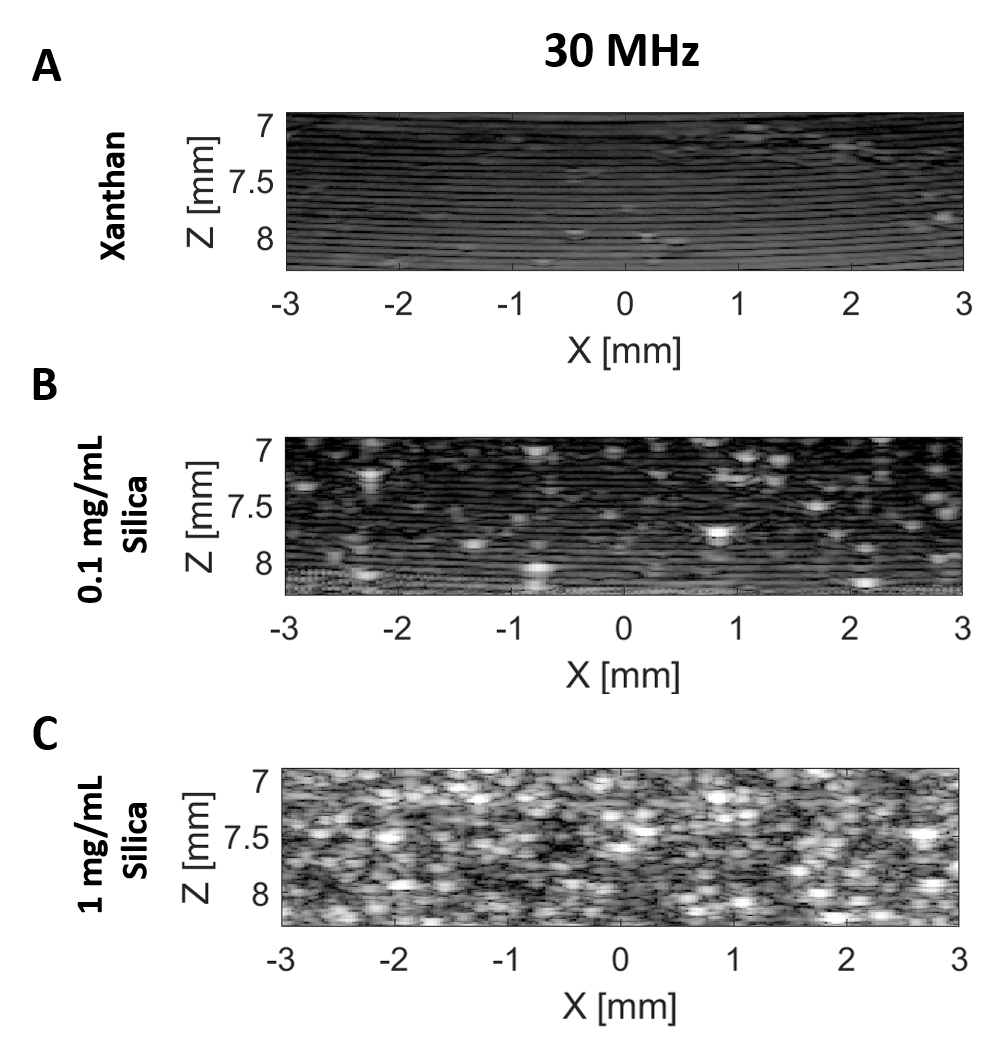

Supplement: S5 Fig — B-mode images at 30 MHz of phantoms with 50-μm layer thickness and (A) only xanthan gum (0.833 mg/mL; Phantom 6), (B) xanthan gum (0.833 mg/mL) and 0.1 mg/mL silica particles (Phantom 7), and (C) xanthan gum (0.833 mg/mL) and 1 mg/mL silica particles (Phantom 8). (TIF) [file pone.0260737.s005.tif]
