# Supplementary material for: Projection-based stereolithography for direct 3D printing of heterogeneous ultrasound phantoms
Source: PLoS One. 2021 Dec 9;16(12):e0260737. doi: 10.1371/journal.pone.0260737 (PMC8659365; doi:10.1371/journal.pone.0260737)
Supplement: S1 Table — (DOCX) [file pone.0260737.s006.docx]

**S1 Table: Percent differences over time of hydrogels stored in PBS at 4°C and 25°C when compared to designed CAD dimensions.**

| **Phantom (Storage Media @ Temperature)** | **Measurement Type (Direction)** | **0 Days After**  **(% Change from CAD)** | **7 Days After**  **(% Change from CAD)** | **14 Days After**  **(% Change from CAD)** | **31 Days After**  **(% Change from CAD)** |
| --- | --- | --- | --- | --- | --- |
| **PEGDA-GelMA 1**  **(PBS @ 4°C)** | Lumen (y) | 7.1±5.6 | 2.5±0.9 | 3.2±1.1 | 0.7±1.0 |
|  | Lumen (z) | 6.8±2.2 | 4.3±1.1 | 4.9±1.1 | 1.6±0.0 |
|  | Body (y) | 6.7±0.3 | 2.1±0.2 | 2.8±0.8 | 1.8±0.2 |
|  | Body (z) | 1.4±0.2 | -2.6±0.2 | -2.4±0.2 | -3.3±1.9 |
| **PEGDA-GelMA 2**  **(PBS @ 4°C)** | Lumen (y) | 5.1±1.1 | 5.8±1.2 | 7.8±1.1 | 15.2±3.9 |
|  | Lumen (z) | 6.9±3.1 | -0.4±1.1 | -2.3±0 | 10.0±3.5 |
|  | Body (y) | 6.3±0.1 | 2.9±0.1 | 2.6±0.1 | 2.4±0.7 |
|  | Body (z) | 1.2±0.4 | -4.3±0.5 | -4.7±0.5 | -3.1±0.9 |
| **PEGDA-GelMA 1**  **(PBS @ 25°C)** | Lumen (y) | 3.9±0.1 | 8.0±0.2 | 10.5±4.4 | 12.4±2.3 |
|  | Lumen (z) | 1.6±0.0 | 2.5±0.9 | 1.0±1.1 | 5.5±3.4 |
|  | Body (y) | 6.1±0.1 | 1.4±3.4 | 2.4±1.2 | 6.8±1.4 |
|  | Body (z) | 1.4±0.5 | -6.1±2.7 | -5.0±1.0 | -0.6±0.9 |
| **PEGDA-GelMA 2**  **(PBS @ 25°C)** | Lumen (y) | 6.8±2.9 | 7.7±2.0 | 11.1±1.0 | 6.6±2.4 |
|  | Lumen (z) | 7.7±2.1 | 3.5±1.9 | 2.9±1.0 | 10.1±3.1 |
|  | Body (y) | 6.7±1.2 | 2.7±0.7 | 1.5±0.3 | 5.6±0.4 |
|  | Body (z) | 1.3±0.9 | -6.4±0.5 | -7.5±0.8 | 4.9±0.3 |
| **PEGDA 1**  **(PBS @ 4°C)** | Lumen (y) | 21.5±5.9 | 39.5±1.8 | 31.9±8.2 | 45.0±7.7 |
|  | Lumen (z) | 4.2±14.1 | 28.7±3.1 | 13.4±3.4 | 17.1±16.1 |
|  | Body (y) | 13.6±2.8 | 26.4±1.2 | 25.5±0.3 | 28.0±1.0 |
|  | Body (z) | 5.9±7.2 | 16.0±6.8 | 10.3±2.0 | 11.6±3.7 |
| **PEGDA 2**  **(PBS @ 4°C)** | Lumen (y) | 27.4±9.0 | 42.5±3.7 | 29.4±2.0 | 51.9±6.4 |
|  | Lumen (z) | 5.5±6.7 | 23.8±3.8 | 14.0±10.8 | 17.3±4.3 |
|  | Body (y) | 15.5±2.6 | 24.9±0.5 | 22.5±1.0 | 26.7±1.6 |
|  | Body (z) | 5.5±6.8 | 13.1±2.7 | 7.7±0.6 | 11.1±1.1 |
| **PEGDA 1**  **(PBS @ 25°C)** | Lumen (y) | 28.7±3.0 | 38.9±6.4 | 31.4±1.8 | 47.2±4.3 |
|  | Lumen (z) | 4.2±10.8 | 21.9±8.9 | 12.7±8.8 | 19.4±11.1 |
|  | Body (y) | 13.0±1.5 | 20.2±0.5 | 14.7±1.0 | 19.7±0.5 |
|  | Body (z) | 1.8±7.1 | 3.7±3.3 | -0.1±2.9 | 1.1±2.2 |
| **PEGDA 2**  **(PBS @ 25°C)** | Lumen (y) | 30.0±5.9 | 38.3±5.6 | 28.1±8.6 | 44.6±4.3 |
|  | Lumen (z) | 4.9±11.3 | 17.1±9.3 | 11.1±7.5 | 16.7±4.0 |
|  | Body (y) | 15.3±2.7 | 2.05±0.5 | 16.8±0.8 | 18.4±0.1 |
|  | Body (z) | 8.2±8.9 | 8.4±6.8 | 1.4±1.7 | 1.5±2.0 |
