# Supplementary material for: Projection-based stereolithography for direct 3D printing of heterogeneous ultrasound phantoms
Source: PLoS One. 2021 Dec 9;16(12):e0260737. doi: 10.1371/journal.pone.0260737 (PMC8659365; doi:10.1371/journal.pone.0260737)
Supplement: S2 Table — (DOCX) [file pone.0260737.s007.docx]

**S2 Table: Results from US elasticity imaging**

| **Phantom Number** | **Cure Time (sec)** | **Shear Wave Velocity (m/s)** | **Young’s Modulus (kPa)** |
| --- | --- | --- | --- |
| **12** | 5 | 1.6±0.1 | 7.6±0.7 |
|  | 7.5 | 2.2±0.1 | 14.3±0.8 |
|  | 10 | 2.3±0.1 | 15.2±0.7 |
|  | 12.5 | 2.3±0.1 | 15.6±0.6 |
| **13** | 8 | 4.9±0.1 | 72.9±1.1 |
|  | 12 | 6.2±0.1 | 115.3±1.1 |
|  | 17 | 6.3±0.1 | 118.9±0.8 |
|  | 20 | 6.4±0.1 | 123.6±0.9 |
|  | **Imaging Angle** | **Shear Wave Velocity (m/s)** | **Young’s Modulus (kPa)** |
| **14** | 0º | 2.6±0.1 | 20.3±0.7 |
|  | 45º | 2.5±0.04 | 18.6±0.5 |
|  | 90º | 2.3±0.03 | 15.6±0.4 |
