# Supplementary material for: Projection-based stereolithography for direct 3D printing of heterogeneous ultrasound phantoms
Source: PLoS One. 2021 Dec 9;16(12):e0260737. doi: 10.1371/journal.pone.0260737 (PMC8659365; doi:10.1371/journal.pone.0260737)
Supplement: Attachment — Submitted filename: Comments on Manuscript Submitted.docx [file pone.0260737.s008.docx]

Comments on Manuscript Number PONE-D-19-07361
Projection-based stereolithography for direct 3D printing of heterogeneous ultrasound phantoms

We would like to thank the editor for arranging the review of our manuscript and the referees for their helpful comments. We feel that the suggestions and comments from each reviewer make the manuscript clearer and more scientifically robust. We addressed all reviewer comments and suggestions (provided below in red). The changes made to the manuscript in direct response to reviewer concerns are provided below in quotes, while we took the advice of both reviewers and attempted to consolidate and clarify the text throughout the manuscript.

Reviewer #1

This manuscript reports a survey of methods to creating “3D printed” hydrogel materials for use as ultrasound phantoms. The manuscript states that there is a lack of anthropomorphic ultrasound phantoms and attempts to demonstrate the ability to address key issues needed in ultrasound phantoms of current interest to the investigators. A wide variety of tasks, such as varying the “echogenicity” of the material, varying the overall stiffness of the material, and creating channels within the material to simulate vasculature, were attempted.

The authors should clearly declare whether they are trying to mimic a particular tissue type, or if they are simply investigating the range of phantom properties that can be created with this approach. There was an attempt to quantify the acoustic properties of these materials, but the lack of rigor in that effort is one of the weaknesses of the manuscript.

There is great merit in the effort presented here, but more work needs to be done, and more rigor is needed in the quantification of material properties to argue that these 3D printed materials are viable alternatives to existing materials.

The goal of the manuscript was to present a method for creating US phantoms which can generally mimic tissue and can be further customized for a variety of applications; the work of demonstrating that these hydrogel materials can mimic ultrasound properties of tissue has already been demonstrated in the cited reference by Aliabouzar *et al*. In our submission, we comment on relative echogenicity of phantom properties when describing sections within phantoms.

*“Collectively, these results show that projection-based stereolithography allows for easy, scalable, and customizable fabrication of complex ultrasound phantoms whose properties can be established to mimic those of native tissue.” (Line 35)*

*“Ultimately, our method provides researchers with the ability to develop phantoms with customized backscatter and elasticity values as well as custom vasculature such that they may be made sufficiently realistic and detailed to generally mimic tissue to the degree necessary for further development and validation of more advanced US imaging techniques, such as those being championed by the QIBA initiative.” (Line 127)*

**General Comments**

1. The authors argue that their 3D printed materials are created “in a single step”. However, each ‘step’ in their process produces a thin layer (50—200 micrometers) of material. That ‘step’ must be repeated many times over to create any reasonable volume. The authors perspective on phantom creation should be recalibrated.

We agree that the use of the term “step” in this context is confusing given that a stepper motor is used to control extrusion for each print layer. This term has been replaced with “print session” throughout the paper in an effort to improve manuscript clarity.

*“Using a 3D-printing technique based on stereolithography, we fabricated ultrasound phantoms using soft materials in a single print session, without the need for material casting or back-filling.” (Line 26)*

2. The authors argue that there is a lack of ultrasound phantoms configured in shapes similar to real tissues. They should search for the combined terms "anthropomorphic", "ultrasound", and "phantoms". It seems they would be surprised by the thousands of results they would find. This would include phantoms built from vascular casts of real organs (though clearly not down to the level of capillaries). If, on the other hand, they would refute the degree to which those phantoms are "anthropomorphic" they need to demonstrate phantoms that exceed current capabilities prior to publication.

We would like to thank the reviewer for his concern regarding the focus of the paper. The manuscript contains references to multiple current anthropomorphic US phantom techniques and outlines how this work differs from these previously existing phantoms, in either quality, anthropomorphic validity, or rigor of fabrication. However, we were not clear in regards to why our method was a better choice for fabrication of US phantoms. We have clarified the introductory text to make this clearer.

*“Ultrasound (US) imaging has long been a valuable tool for medical diagnostics due to its non-invasive nature, high resolution, dynamic-imaging capabilities, and its capacity to assess tissue properties beyond simple anatomy (e.g., tissue stiffness) [1]. The use of US-based quantitative imaging biomarkers (QIB) – such as assessing volume blood flow (VBF), tissue perfusion with contrast-enhanced US (CEUS), or shear wave speed (SWS) elasticity imaging – offers tremendous potential in providing more effective, patient-specific, rational clinical care [2,3]. However, translating QIB methods from research tools to clinical practice has proven challenging, in large part because the imaging phantoms needed to support robust quality assurance (QA) programs for these modalities are insufficient. Given the strong dependence of QIBs on the specific functional (e.g., blood flow) and anatomical (e.g., vessel topology) aspects of an interrogated biological system, current imaging phantoms – due to their overly “simplistic design” – do not adequately simulate the wide range of structural complexities and biological variation inherent in a human subject, thus leading to overestimated measurements for precision, particularly reproducibility, and inaccurate assessment of bias [4–7]. Studies have noted that many physiological (e.g., vessel permeability) and anatomical (e.g., vessel scale and dimensionality) characteristics are not accurately replicated in current imaging phantoms [8,9].*

*The RSNA QIB Alliance (QIBA) Metrology Working Group recently warned that “phantoms do not represent the complexity of human targets; thus, precision is often overestimated” [10]. Consequently, they claimed that “improved realism of phantoms [is an] area worthy of further investment” [10]. An improved phantom platform is critical for validation and optimization of more established US-based QIB methods (e.g., VBF, CEUS, & SWS), while such a platform would also be of tremendous value in the development of newer QIB approaches, such as super-resolution imaging, acoustic angiography, or photoacoustic-ultrasonic oxygen saturation (PAUS sO_2_) imaging [11–13]. To meet these needs, the next-generation imaging phantom platform should be capable of capturing the scale, tortuosity, density, and functionality of vasculature and of emulating the tissue backscatter heterogeneity, viscoelasticity, and anisotropy that is characteristic of human biology.*

*Although homogeneous tissue-mimicking phantoms made of hydrogels, rubbers, and other tissue-mimicking materials are widely available, both commercially (e.g., CIRS, Nuclemed, True Phantom) and otherwise, most provide only gross organ-level anatomy- and physiology-mimicking properties [14–18]. While this level of tissue-mimicking is sufficient for routine imaging and machine QA testing, these phantoms ultimately lack the heterogeneity and functional aspects (e.g., realistic blood flow) necessary for use with US-based QIB imaging. Tissue-mimicking US phantoms generally should contain regions of spatially varying acoustic backscatter, generated from mismatching acoustic impedance due to local changes in density and/or bulk modulus [19–22]. Additionally, phantoms for Doppler imaging and elasticity imaging should include flow-supporting channels and regions of varying stiffness, respectively. Unfortunately, there currently exist limitations in fabricating realistic and well-characterized phantoms with the degree of spatial control necessary to make voxel-specific changes in the desired phantom properties.” (Line 40)*

*“Indeed, multiple groups have used 3D printing to develop custom molds for generating US phantoms that mimic patient anatomy. As an example, fused deposition modeling (FDM) has been used to fabricate plastic molds for casting US phantoms of the thyroid [29], a fluid-flow phantom [30], and phantoms with bone-like inclusions to mimic the spine [31] and rib cage [32]. This process can also be used to create molds of individual tissue-mimicking components with varying levels of backscatter, which are then combined to produce US phantoms mimicking whole organs, such as the human heart or placenta [33]. Though these groups have successfully generated anatomically relevant phantoms with complex geometries and varying levels of backscatter, the processes inherently require significant time and effort while limiting the ultimate phantom complexity (i.e., variation in sub-voxel structural, acoustic, and stiffness properties) that is reasonably achievable.” (Line 91)*

*“Ultimately, our method provides researchers with the ability to develop phantoms with customized backscatter and elasticity values as well as custom vasculature such that they may be made sufficiently realistic and detailed to generally mimic tissue to the degree necessary for further development and validation of more advanced US imaging techniques, such as those being championed by the QIBA initiative.” (Line 127)*

3. The methods for estimation of acoustic attenuation and backscatter are not clear. For example, the (frequency domain) units reported for the attenuation coefficients are not consistent with the (time domain) methods used to obtain that estimate. Further, no details are provided for quantitative assessment of backscatter. There are a variety of methods in the literature for estimating acoustic backscatter coefficients, and estimates of that property in biological media, but the methods used for characterizing the 3D printed materials are not described.

Given the aforementioned issues brought forth by the reviewer, we have removed the attenuation characterization portion of the manuscript and have changed the section title of this section to read as follows:

*“****Assessment of sound speed” (Line 332)***

Regarding the reviewer’s concerns regarding the estimation of backscatter, it was not our intention to classify the absolute backscatter coefficient of these materials; instead we measured the relative change in the amount of backscatter via analysis of single-channel data and CNR measurements for the printed targets (i.e., increased echogenicity) relative to a phantom’s background. We have changed references to US backscatter measurement to assessment of US properties throughout the manuscript, such as the following:

*“****Assessment of US properties***

*To investigate the US properties of the printed phantoms, we first acquired 3D US data of phantoms with different print-layer thicknesses (Phantoms 1, 2; Fig 2A) using 12, 30, and 50 MHz imaging transmit frequencies.” (Line 302)*

4. It seems that there is a missed opportunity here. The authors demonstrate the ability to manufacture phantoms with periodic scattering structures that can be anisotropic. Such a material might reasonably mimic the scattering properties of muscle or tendon, or other resolvable or subresolution periodic scattering, where no other material combination has been demonstrated (in extended volumes). The authors might address this possibility.

We would like to thank the reviewer for suggesting such a great (and possible) idea. While we had considered this idea in the original submission, we did not make that clear in the body of the text. We have added the following points to state such a possible application more clearly:

*“The base photocure time of the phantom was 5 seconds, while columns running throughout the phantom in a checkerboard pattern (to mimic periodic scattering structures such as muscle fibers) were cured for a total time of 7.5 seconds to induce transverse anisotropy (Fig 2D).” (Line 253)*

*“Further development of this phantom model could prove useful for modeling tissues with striated fibers such as muscle, as this phantom contained periodic anisotropic, chord-like structures which could mimic naturally occurring fiber orientation.” (Line 503)*

5. There is a lack of information on the materials used in this study. How dependent are these phantoms on the source materials used in their production? See also the related specific comments below.

The materials used in this study were based on a prior paper from our group, which had been accepted but not yet published upon submission of this paper. Full details can be found in citation by Grigoryan *et al.* (which was not accepted for publication prior to our initial submission) and is now provided in the manuscript as follows:

*“We previously used a pSLA technique developed to print binary structures using poly(ethylene glycol) (PEG)-based hydrogels [39].” (Line 118)*

*“US phantoms were fabricated using a pSLA system, as described previously [39].” (Line 127),* where reference 39 now reads as:

*39. Grigoryan B, Paulsen SJ, Corbett DC, Sazer DW, Fortin CL, Zaita AJ, et al. Multivascular networks and functional intravascular topologies within biocompatible hydrogels. Science. 2019;364: 458–464. doi:10.1126/science.aav9750*

6. Are the images in figure 4 representative examples of the (lack of) uniformity of these materials? If so, what are the prospects for printing materials consistent with the uniformity of commercially-available ultrasound phantoms?

Some of the non-uniformities seen in phantom images in Figure 4 were a result dust settling on the top of the phantom after being printed. Given that the surface was not perfectly parallel, this region was included in the C-scan slice during the original image formation. We have since adjusted the C-scan plane to exclude this (phantom) surface region, which has improved image quality.

**Specific Comments**

1. The authors state “We previously used a pSLA technique developed in our laboratory to print binary structures using poly(ethylene glycol) (PEG)-based hydrogels” and then cite work other than theirs. Is that correct? If so, the context of that citation needs to be clarified.

As previously mentioned, the correct citation for our production technique is as follows:

*“Grigoryan B, Paulsen SJ, Corbett DC, Sazer DW, Fortin CL, Zaita AJ, et al. Multivascular networks and functional intravascular topologies within biocompatible hydrogels. Science. American Association for the Advancement of Science; 2019; 364: 458–464. doi:10.1126/science.aav9750”*

2. The authors state “Our semi-automated printing approach can produce phantoms at rates of up to 3 cm/hr, improving the consistency and efficiency of phantom fabrication over other techniques.” This seems significantly more time consuming that current phantom casting methods. It is also not clear how this printing rate affects ‘consistency’. The authors should clarify their statement.

We agree with the reviewer that this statement lacks clarity and have thus updated the text as follows:

*“Our semi-automated approach can print at rates of up to 3 cm (in the Z axis) per hour, permitting the timely production of fully customized and cured imaging phantoms.” (Line 125)*

3. The authors state “All gels were allowed to swell to equilibrium overnight in deionized (DI) water prior to testing.” Was this swelling process controllable? If so, how? If not, how are these materials repeatably reproduced? Please add some detail about this step and its results.

While swelling does occur due to rehydration driven by osmosis and is repeatable under matched conditions, the swelling is not the only purpose for the overnight soak. In order to reflect this, we have changed the statement in the text to read as follows:

*“All gels were allowed to rehydrate to equilibrium overnight in deionized (DI) water prior to testing. This rehydration process also flushed any unreacted solution from vessel lumen and washed the phantom of tartrazine to provide transparent media.” (Line 180)*

4. Line 172: The authors state “...these printed targets were too organized to fully developed speckle...”. The grammar needs to be corrected.

We thank the reviewer for identifying this error. This sentence has been fixed to now read:

*“...these printed targets were too organized to generate fully developed speckle...” (Line 196)*

5. The authors state that they added silica particles to manipulate the scattering properties of the phantom materials. Please provide details on exactly what ‘silica’ particles are used. What is their source, their diameter distribution, etc., and why was this specific material selected.

Information regarding the source, size, and justification for using silica particles has now been provided. This portion of the Methods now reads as follows:

*“Therefore, we fabricated several phantoms (phantoms 8, 15, and 16) with varied concentrations of 40-μm diameter silica particles (MIN-U-SIL-40, U.S. Silica Co., Mill Creek, OK) to provide tissue-mimicking US speckle, as has been demonstrated in previous work [14].” (Line 198)*

6. Xanthan gum was added to some materials in an effort to deal with gravitational settling of the silica particles. But it seems that there was no investigation into the effects on the material properties by adding this gum. For example, did it change the sound speed, attenuation, shear wave speed or its dispersion?

Xanthan gum was added to samples containing silica, as well as one standalone phantom to test the effect on signal and CNR. While Xanthan gum was not investigated for changes in the sound speed, attenuation, or elasticity data, it was shown to not have an effect on US signal or CNR when compared to the PEGDA alone. It was additionally added to the 3D flow phantom to trap silica in suspension but had no effect on the vessels. In future studies, it could surely be warranted to investigate the effect of Xanthan gum on sound speed as well as shear wave properties. We have updated the text to reflect regions where Xanthan gum was compared to bare PEGDA phantoms.

*“This second set of phantoms was fabricated using the same 20 wt% 6 kDa PEGDA pre-polymer formulation, though we tested two additional phantom compositions with added xanthan gum (0.83 mg/mL), with and without 0.1 mg/mL silica particles added, all at 50-µm layer thickness (Phantoms 6-8; Fig 2B).” (Line 233)*

*“This increase in US backscatter signal within the targets was noted at all three investigated imaging frequencies, and in phantoms with and without Xanthan gum or silica (Phantoms 6-8).” (Line 438)*

*“In addition, the silica particles included in Phantom 8 provided speckle and resulted in a lower standard deviation within the ROIs, while Xanthan gum alone (Phantom 7) caused no noticeable change compared to the bare hydrogel phantom (Phantom 6).” (Line 446)*

7. Equation 1: Shouldn’t the denominator be the square root of the sum of variances?

Yes, the reviewer is absolutely correct, and we apologize for the typo, which has since been corrected. All of the original analysis, however, was done with the correct (and now depicted) form of the CNR expression, as follows:

$CNR=\frac{\mu_{signal}-\mu_{background}}{\sqrt{{\sigma_{signal}}^{2}+{\sigma_{background}}^{2}}}$, (Line 325)

8. A different method was used to create a phantom for estimating sound speed of the material. How do the authors know whether printing or curing affect sound speed? Without knowledge of sound speed, how do they know how long the data segments were for attenuation estimation

The purpose of the speed-of-sound study was to characterize the bulk speed of our hydrogel formulation, which was consistent with values measured by Aliabouzar *et al*. We agree that it could be warranted to investigate the effect of print layers on sound speed in future work. As our attenuation study was removed from this work, we have not addressed the second concern but will take it into account for attenuation measurement in future work.

*“***Assessment of sound speed**

*Using Phantom 9, we characterized the speed of sound through our hydrogel formulation to be 1490±5 m/s, which is slightly lower than that expected for soft tissue in general but is approximately equal to the speed of sound in fat [54]. While this was conducted in a molded phantom to provide a homogenous medium over sufficient imaging depth to facilitate accurate measurements, in future work we will investigate this parameter further in phantoms with printed layers, as well as varying density and elasticity to determine the full range of phantom sound speed. This result is similar to work done by Aliabouzar et al., which reported sound speed values between 1500-1600 m/s for the same class of hydrogel [40]. With subtle modifications, these phantoms could likely be made to mimic the sound speed of a broad spectrum of human tissue [54].” (Line 448)*

*“In order to measure the speed of sound in the pSLA-printed material, we cast a large, uniform rectangular phantom of a known thickness (measured using calipers) with no inclusions or printed layers (i.e., a homogeneous block; Phantom 9).” (Line 333)*

9. The authors created a small phantom for estimation of shear wave speeds. What were the dimensions of the phantom? Was there any potential for Lamb wave propagation? Could this phantom be considered a semi-infinite medium (so that the wave is traveling far away from any boundaries, as needed for the simple approach to modulus estimation)?

We would like to thank the reviewer for his concerns about this phantom. We have clarified the phantom dimensions, which can be found in the text of the methods as well as in Figure 2, to be 35 x 20 x 10 mm. As stated in the text, we are assuming that the phantoms provide a linear, isotropic, elastic medium when calculating Young’s moduli. We have also added the following statement to the text to address the reviewer’s concern concerning Lamb wave propagation, which would manifest as (measurable) surface displacement following the radiation force excitation:

“No significant displacement was noted on the surface of either phantom.” (Line 494)

10. Lines 384—392 are a description of the methods used, not results, and should be moved to the appropriate section of the manuscript.

We apologize for this oversite. A portion of this text has since been incorporated into the Materials and Methods section, while a portion was eliminated to avoid any possibility of redundancy.

11. The authors comment on the flow through 60 micron diameter channels, and that those are sometimes not clear channels. That suggests there should be some comment, if not quantification, of the surface of these channels. Rough printed surface would affect the flow dynamics.

We apologize for this section being unclear. Yes, because of the physics of photocuring through the lumen, channels will certainly not always be symmetric in nature and may have topologies that are not circumferentially uniform. To elucidate this point, we have rephrased the text as follows:

*“The channel diameters were inconsistent throughout the extent due to differences in light scattering during photocuring, but diameters were measured as small as 60 µm (as measured by B-mode US imaging; Fig 5A). Despite this inconsistency, all channels supported flow that tended to be laminar in nature upon investigation with Doppler imaging*.” (Line 509)

Reviewer #2

1. The projection-based stereolithography method described in this manuscript to produce ultrasound phantoms has been published---or is in the process of revision as reference [40]. As I do not have access to that manuscript other than the title, "Functional intravascular topologies and multivascular networks within biocompatible hydrogels", I can only assume that a fair amount of overlap exists. Certainly the other references detail the stereolithography, and so I am left wondering what is in that other manuscript, a difficult matter as it leaves me with little choice but to recommend at least major revision to include that manuscript as a part of this one's assessment for this journal.

We apologize for the incomplete citation. The manuscript to which the author is referencing had just been conditionally accepted at the time of this submission; however, it was not yet available online and thus its full citation had yet to be established. This manuscript has now been made available online and given a complete citation, which is provided now in the manuscript [39] and below:

Grigoryan B, Paulsen SJ, Corbett DC, Sazer DW, Fortin CL, Zaita AJ, et al. Multivascular networks and functional intravascular topologies within biocompatible hydrogels. Science. American Association for the Advancement of Science; 2019; 364: 458–464. doi:10.1126/science.aav9750

2. There are ample sources, commercial and otherwise, of tissue-mimicking ultrasound phantoms. Offhand, Computerized Imaging Reference Systems dominates this industry, while Nuclemed (Belgium) and TruePhantom are another two commercial suppliers, and the effort over the past few years has been to provide multiple schemes of accurately representing tissue, for example, ultrasound and MRI. While the use of stereolithography to produce the results reported here is mildly interesting, it isn't especially useful in my opinion.

We would like to thank the reviewer for bringing up commercial phantom options, which we have acknowledged in the text. In regards to the usefulness of the phantoms generated in this work, we have updated the introduction to clarify why we believe current phantom options are not sufficient for many emerging applications. While the phantoms mentioned by the reviewer are sufficient for routine machine QA and testing, they are generally lacking in complexity in either vessels or micro-scale heterogeneity which can lead to issues in imaging, for example, quantitative imaging biomarkers (QIB). With this in mind, the phantoms presented in this work are a necessary building blocks toward phantoms sufficient for QIB imaging in that they demonstrate the ability to fabricate phantoms which can provide realistic vasculature along with voxel-specific backscatter and viscoelasticity properties, along with anisotropy. We have made several updates to the text as follows:

*“Modern diagnostic imaging is continuing to increase its clinical impact, particularly with the introduction of ultrasound-based quantitative imaging biomarkers (QIB), with offer tools to more precisely identify and track various pathologies. However, current ultrasound phantom designs are generally too simplistic to accurately simulate the biologic variability within a human. Therefore, there is a need to create a platform that is capable of generating well-characterized phantoms that can mimic the basic anatomy, function, and mechanical properties of native tissues.” (Line 1)*

*“Ultrasound (US) imaging has long been a valuable tool for medical diagnostics due to its non-invasive nature, high resolution, dynamic-imaging capabilities, and its capacity to assess tissue properties beyond simple anatomy (e.g., tissue stiffness) [1]. The use of US-based quantitative imaging biomarkers (QIB) – such as assessing volume blood flow (VBF), tissue perfusion with contrast-enhanced US (CEUS), or shear wave speed (SWS) elasticity imaging – offers tremendous potential in providing more effective, patient-specific, rational clinical care [2,3]. However, translating QIB methods from research tools to clinical practice has proven challenging, in large part because the imaging phantoms needed to support robust quality assurance (QA) programs for these modalities are insufficient. Given the strong dependence of QIBs on the specific functional (e.g., blood flow) and anatomical (e.g., vessel topology) aspects of an interrogated biological system, current imaging phantoms – due to their overly “simplistic design” – do not adequately simulate the wide range of structural complexities and biological variation inherent in a human subject, thus leading to overestimated measurements for precision, particularly reproducibility, and inaccurate assessment of bias [4–7]. Studies have noted that many physiological (e.g., vessel permeability) and anatomical (e.g., vessel scale and dimensionality) characteristics are not accurately replicated in current imaging phantoms [8,9].*

*The RSNA QIB Alliance (QIBA) Metrology Working Group recently warned that “phantoms do not represent the complexity of human targets; thus, precision is often overestimated” [10]. Consequently, they claimed that “improved realism of phantoms [is an] area worthy of further investment” [10]. An improved phantom platform is critical for validation and optimization of more established US-based QIB methods (e.g., VBF, CEUS, & SWS), while such a platform would also be of tremendous value in the development of newer QIB approaches, such as super-resolution imaging, acoustic angiography, or photoacoustic-ultrasonic oxygen saturation (PAUS sO_2_) imaging [11–13]. To meet these needs, the next-generation imaging phantom platform should be capable of capturing the scale, tortuosity, density, and functionality of vasculature and of emulating the tissue backscatter heterogeneity, viscoelasticity, and anisotropy that is characteristic of human biology.*

*Although homogeneous tissue-mimicking phantoms made of hydrogels, rubbers, and other tissue-mimicking materials are widely available, both commercially (e.g., CIRS, Nuclemed, True Phantom) and otherwise, most provide only gross organ-level anatomy- and physiology-mimicking properties [14–18]. While this level of tissue-mimicking is sufficient for routine imaging and machine QA testing, these phantoms ultimately lack the heterogeneity and functional aspects (e.g., realistic blood flow) necessary for use with US-based QIB imaging.” (Line 40)*

3. The production of the phantoms in this study could not be really claimed to be in "a single step" as the phantoms are produced layer-by-layer.

We agree with both reviewers that the use of the term “step” in this context is confusing given that a stepper motor is used to control extrusion for each print layer. This term has been replaced with “print session” throughout the manuscript in an effort to improve manuscript clarity.

4. Another worry is the claim that this process produces phantoms that "mimic the anatomy and mechanical properties of native tissues". While I can see the utility of the phantoms as produced, as far as I can see there is no data to support the statement that these materials mimic real tissue. If one were to have a comparison of the two---real tissue and its stereolithography-produced analog---and find they are at least similar, then this claim would be sound.

In our abstract, we originally stated that it is important to have phantoms capable of mimicking the properties of native tissue, and while we still believe this to be true, we have reworded large portions of the abstract and introduction as stated in response to reviewer concern #2. We believe that using this platform, researchers will be able to create phantoms with these capabilities, especially given the range of tissue-mimicking ultrasound properties characterized by Aliabouzar *et al*. for our class of hydrogel. Therefore, we have added several updates to the text to reinforce the point that we are providing a platform which is capable of generally mimicking multiple tissue types for multiple US modalities, and we have added several points of emphasis to the text to clarify this as follows:

*“Collectively, these results show that projection-based stereolithography allows for easy, scalable, and customizable fabrication of complex ultrasound phantoms whose properties can be established to mimic those of native tissue.” (Line 35).*

*“Ultimately, our method provides researchers with the ability to develop phantoms with customized backscatter and elasticity values as well as custom vasculature such that they may be made sufficiently realistic and detailed to generally mimic tissue to the degree necessary for further development and validation of more advanced US imaging techniques, such as those being championed by the QIBA initiative.” (Line 127)*

*“While this class of hydrogel has been shown to generate material with US properties (i.e., speed of sound, attenuation) generally in the range of human tissues [40], this previous work did not explore formulations and fabrications designed exclusively for the purpose of making the specimen amenable for high-quality, clinical US imaging. In this work, we incorporated an additional functionality into our existing 3D-printing technique to vary the levels of light exposure within each printed layer, thereby controlling the resulting US backscatter and stiffness” (Line 119)*

*“Ultimately, we have demonstrated the capacity to fabricate monolithic hydrogels containing open-channel networks, regions of varied elasticity, and regions of varied US signal for generating advanced US phantoms with tissue-like properties in a single, semi-automated process.” (Line 565)*

5. It seems to me the effort expended in producing this voluminous manuscript is too broad and without enough focus to produce results the reader could make use of in assessing whether the statements made in the abstract and conclusions are valid or not. In some ways I have the feeling this manuscript is two or three manuscripts in waiting, with only the depth required to secure the publication of those manuscripts.

We appreciate the criticism from the reviewer; upon reflection, we agree that the scope of the manuscript was too broad and have significantly edited the manuscript. We have removed extraneous information as well as some testing which we believe was not essential to demonstrate the potential benefit of our phantom platform. We have additionally moved one of the tables to the supplemental section to not crowd the body of the text.
