# Supplementary material for: Projection-based stereolithography for direct 3D printing of heterogeneous ultrasound phantoms
Source: PLoS One. 2021 Dec 9;16(12):e0260737. doi: 10.1371/journal.pone.0260737 (PMC8659365; doi:10.1371/journal.pone.0260737)
Supplement: Attachment — Submitted filename: Reviewer Response_FINAL.docx [file pone.0260737.s009.docx]

*We would like to thank the editor for arranging the review of our manuscript and the referees for their helpful comments. We have addressed all reviewer comments and suggestions and feel that the quality and clarity of our manuscript has improved as a result. Reviewer comments are provided verbatim in bold text, and our reply to each comment is given immediately following with excerpts from the revised manuscript provided in quotations. Note that the line numbers provided for these excerpts have been updated and may vary from those noted by reviewers from the prior submission.*

***Reviewer #1:***

**The goal of the reported phantom work is relevant to quantitative ultrasound methods. Its main innovation is to demonstrate a single-session 3D printing approach for a variety of ultrasound applications that may simplify phantom development and allows control of backscatter and stiffness at the voxel level.**

**The presentation is not focused, however, rather several different phantom designs for different potential US applications are demonstrated but the rationale for these designs is not offered.**

**Line 53. It may help to clearly state what is the purpose of having complex phantoms that approximate anatomy, function and mechanical properties of real tissues. How would they be used for QIB development? How close is good enough? The data presented do not appear to actually address anthropomorphic organ-mimicking phantoms anyway. Clearly state what they add that cannot be accomplished with simpler QIB phantoms that permit isolation of tissue properties independently or in simpler combinations.**

We have reorganized and edited the manuscript to make it more focused and concise. Even with the inclusion of a requested phantom stability and precision experiment, we were able reduce the overall word count of the manuscript by about 1,000 words. We have also ensured rationale for the presented phantom designs is provided in the text:

Line 39. “In addition to traditional anatomy-based US applications, the use of US-based quantitative imaging biomarkers (QIB) – such as assessing volume blood flow (VBF), tissue perfusion with contrast-enhanced US (CEUS), or shear wave speed (SWS) elasticity imaging – offers tremendous potential in providing more effective, patient-specific, rational clinical care [2,3]. However, translating QIB methods from research tools to clinical practice has proven challenging, in large part because the imaging phantoms needed to support robust quality assurance (QA) programs for these modalities are often insufficient. Given the strong dependence of QIBs on the specific functional (e.g., blood flow) and anatomical (e.g., vessel topology) aspects of an interrogated biological system, current imaging phantoms often do not adequately simulate the wide range of structural complexities and biological variation inherent in a human subject due to their overly “simplistic design.” This leads to overestimated measurements for precision, particularly reproducibility, and inaccurate assessment of bias [4–7]. Studies have noted that many physiological (e.g., vessel permeability) and anatomical (e.g., vessel scale and dimensionality) characteristics are not accurately replicated in current imaging phantoms [8,9]. The RSNA QIB Alliance (QIBA) Metrology Working Group recently warned that “phantoms do not represent the complexity of human targets; thus, precision is often overestimated” [10]. Consequently, they claimed that “improved realism of phantoms [is an] area worthy of further investment” [10]. To this end, an improved phantom platform is critical for validation and optimization of more established US-based QIB methods (e.g., VBF, CEUS, & SWS), while such a platform would also be of tremendous value in the development of newer QIB approaches, such as super-resolution imaging, acoustic angiography, or photoacoustic-ultrasonic oxygen saturation (PAUS sO_2_) imaging. To meet these needs, the next-generation imaging phantom platform should be capable of capturing the scale, tortuosity, density, and functionality of vasculature and of emulating the tissue backscatter heterogeneity, viscoelasticity, and anisotropy that is characteristic of human biology.”

**Most appear to be intended for high frequency US imaging (still a small part of ultrasonography) but this is also not stated.**

High-frequency US was used in many of the studies because of its improved resolution, which is useful for more fundamental analysis of voxel-specific (or layer-specific) attributes of the test phantoms. However, the print-voxel size used in this work is best suited for clinical frequencies (i.e., <12 MHz), in which case the print resolution is significantly smaller than the imaging-voxel size, to ensure volume averaging of the print layers. This point has now been explicitly made in the manuscript:

Line 377. “For phantoms printed with 50-µm layers, the print-layer boundaries were spatially averaged even using a 50-MHz center frequency, and they were indistinguishable at 12 MHz (Fig S2), indicating that such print-voxel size is best suited for fabricating phantoms intended for more typical clinical US frequencies (i.e., <12 MHz).”

**It is argued that the proposed printing method has advantages over other phantom methods but these advantages are not clearly stated or demonstrated.**

In the Introduction, we have now been sure to clearly discuss the disadvantages of current phantom techniques and the advantages of our phantom platform to address such deficiencies.

Limitations of current phantom fabrication platforms:

Line 64. “Although homogeneous tissue-mimicking phantoms made of hydrogels, rubbers, and other tissue-mimicking materials are widely available, both commercially (e.g., CIRS, Nuclemed, True Phantom) and otherwise*,* most provide only gross, organ-level anatomy- and physiology-mimicking properties [11–15]. While such tissue-mimicking extent is generally sufficient for routine imaging and system QA testing, these phantoms ultimately lack the heterogeneity and functional aspects (e.g., realistic blood flow) ideal for use with US-based QIB imaging. For instance, phantoms for Doppler imaging and elasticity imaging should include flow-supporting channels and regions of varying stiffness, respectively. Unfortunately, there currently exist limitations in fabricating realistic and well-characterized phantoms with the degree of spatial control necessary to make such voxel-specific changes in the desired phantom properties.

To address the traditionally limited geometric complexity in phantoms, researchers have investigated tissue-mimicking phantoms via additions of materials such as tube-like structures and inclusions of varying backscatter and/or stiffness to homogeneous tissue-mimicking phantom bases. However, such processes are generally time and labor intensive (i.e., require multiple casting sessions), are generally compatible with only basic geometries, and often introduce imperfections (e.g., air, other unintended materials) that generate imaging artifacts [16–20]. Similar methodology has been used to create tissue-containing phantoms that are anatomically realistic at both macro- and micro-scales but whose use is generally limited owing to their relative lack of characterization compared with wholly fabricated phantoms [21,22].”

Advantages of our phantom platform:

Line 105. “To improve the quality and ease of fabrication of phantoms for a wide range of US-mediated imaging modalities, we used projection-based stereolithography (pSLA) to fabricate phantoms containing voxel-specific US backscatter, phantoms containing direct-printed targets for elasticity imaging, and phantoms with open-channel networks for high-resolution Doppler imaging. We previously used a pSLA technique developed to print binary structures using poly(ethylene glycol) (PEG)-based hydrogels [33]. While this class of hydrogel has been shown to generate material with US properties (i.e., speed of sound, attenuation) generally in the range of human tissues [34], this previous work did not explore formulations and fabrications designed exclusively for the purpose of making the specimen amenable for high-quality, clinical US imaging. In this work, we incorporated an additional functionality into our existing 3D-printing technique to vary the levels of light exposure within each printed layer and voxel, thereby permitting fine spatial control of US backscatter and stiffness. Our semi-automated approach can print at rates of up to 3 cm (in the Z axis) per hour, permitting the timely production of fully customized and cured imaging phantoms. Ultimately, our method provides researchers with the ability to develop phantoms with customized backscatter and elasticity values as well as with complex vasculature such that they may be made sufficiently realistic and detailed to generally mimic tissue for further development and validation of functional and QIB US imaging techniques.”

**For production of a reference phantom or one mimicking tissue properties, there is a need to demonstrate the stability of phantom properties in terms of effects of room temperature, acoustic radiation force, humidity, time, etc. How reproducible are these properties for duplicate phantoms?**

**Perhaps it would help to focus the studies if measurements could be made to determine how accurately and precisely the intended phantom(s) specifications coded in the control software were rendered in the printed results. This seems to be the key question.**

**The authors argue that their 3D printed methods are more repeatable and reproducible that ordinary methods, one of the hallmarks of the QIBA effort. However, no data showing precision of the phantom properties is provided. What is the precision of their method for the acoustic properties that were measured? Was more than one phantom with the same technique printed and compared?**

**Line 180. Rehydration and “swelling” of the phantom material appear to introduce a potential repeatability problem. Do you have data to support it does not?**

We appreciate this feedback from the reviewer. As the four comments are all inquiring about our ability to produce stable, accurate, precise, and reproducible phantoms, we will respond to all of these comments together. We agree with the reviewer that phantom stability is an important point to address. To this point, in the initial submission US signal and CNR of hydrogel printed features were analyzed over the course of 6 weeks when stored in DI water at 4⁰C:

Line 235. “In addition, because PEGDA degrades via hydrolysis, we assessed the temporal stability of the US signal from the printed targets (i.e., regions with additional cure time) and the base hydrogel (i.e., the region with no additional cure time) over 6 weeks. Matched acquisitions were performed 1, 3, and 6 weeks after the initial time-point to assess imaging temporal stability.”

In order to further address the reviewer’s concerns regarding both phantom stability and print fidelity, we performed a follow-up study with the goal of comparing the accuracy (the percent error between geometric features in the phantom and the CAD design), precision (the standard deviation between measurements of a constant feature at multiple points throughout the same phantom), and reproducibility (the percent difference in geometric features between 2 matched phantoms) of our phantoms over the course of 31 days (stability). In this study, half of the PEGDA phantoms were fabricated with GelMA as an additive to assess its effect on improved fidelity and stability. Two matched phantoms of each composition were stored in PBS at either room temperature or 4⁰C for 31 days.

Line 251. “To assess print stability and fidelity, rectangular phantoms with a 1mm-inner-diameter linear lumen (Phantom 9, 10; Fig 2D) were printed and immediately stored in DI water or PBS at 4° or 25°C. In addition to a standard PEGDA formulation (Phantom 9), a second PEGDA formulation containing GelMA (Phantom 10) was also tested. B-mode volumetric imaging (30 MHz; MS400) was performed on matched pairs of each formulation stored at each temperature, with the phantom lumen positioned perpendicular to the transducer axis for four time-points: 0 (i.e., immediately after printing), 1, 2, & 4 weeks following printing. For each time-point, three imaging slices separated by 3-4 mm were chosen, and the midsection extent in the Y- and Z-axis of the rectangular phantom’s walls and lumen was measured using digital calipers in the VevoLAB analysis package (FUJIFILM VisualSonics, Toronto, ON, Canada). Precision was assessed by the standard deviation of measurements for a constant geometric feature (e.g., lumen) across three locations for the same phantom; reproducibility was assessed by comparing the percent difference in geometric features between two matched phantoms; stability was assessed by tracking the change in geometric features for a given phantom over time; and accuracy (i.e., print fidelity) was assessed by comparing the dimensions of printed GelMA-containing phantoms to their CAD dimensions.”

Stability was improved by storing the phantoms at 4⁰C as compared to 25⁰C. Statistics on stability and reproducibility can be seen in Table S1. Hydrogels containing GelMA and stored in PBS at 4° C were the most accurate when compared to the CAD specifications. Precision metrics remained high in almost all cases (3.0% average), as can be seen in Table S1. Across all conditions, measurements in matched phantoms varied by an average of 2.8% (reproducibility). Although print accuracy was estimated at 4.4%, such a metric can be difficult to characterize as interaction of the phantom with the measurement environment (e.g., PBS) can quickly cause changes from the original print geometry. This additional experimentation revealed that GelMA-free phantoms swell in a stable and repeatable manner, all of which is now detailed in Table S1 and in the text:

Line 403. “Hydrogels containing GelMA (Phantom 10) stored in PBS were morphologically most stable (Table S1), experiencing an average (i.e., for both dimensions of the phantom body and lumen) absolute dimensional change compared to their CAD design of 5.2% (day of print) and 4.8% (31 days after print) at 4°C and 4.4% (day of print; this value was also used as the estimate for accuracy) and 6.6% (31 days after) at 25°C. Hydrogels without GelMA (Phantom 9) were less stable, experiencing an average absolute dimensional change compared to their CAD design of 12.4% (day of print) and 26.1% (31 days after print) at 4°C and 8.2% (day of print) and 21.1% (31 days after print) at 25⁰C. Hydrogels stored in DI water, regardless of storage temperature, immediately experienced significant (>20%) dimensional increases (i.e., both lumen and body) when compared to their initial CAD dimensions. Across all combinations, reproducibility measurements between matched phantoms differed by an average of 2.8%, while the average precision between measurements within the same phantom was 3.0%. Although print accuracy was estimated at 4.4%, such a metric can be difficult to characterize as interaction of the phantom with the measurement environment (e.g., PBS) can quickly cause changes from the original print geometry.”

**The manuscript is more in the form of a demonstration report or descriptive technical note rather than a succinct research paper. A number of fabrication factors that affect acoustic properties were examined but accuracy and precision of the measurements are not reported.**

As detailed in the previous comment, accuracy and precision of our phantom fabrication technique have been added to the manuscript. Per PLOS ONE, a “*Research Article* reports the results of original primary research, including quantitative and qualitative studies, methods and software studies, systematic reviews, and other work.” We believe that this is an accurate description of our work.

**What is the limit on spatial accuracy of the printing process?**

The limit on spatial accuracy in the printing process is dependent on the projector used. This work utilized two different projectors. The projector utilized for the majority of the phantoms in this study provides 50-μm print resolution in the X-Y direction, while Phantom 14 (containing small channels) utilized a projector capable of 10-μm resolution. This is now explicitly detailed in the text:

Line 140. “Briefly, this 3D printing system consisted of a Z-stage coated with Polydimethylsiloxane (PDMS) to avoid PEGDA adhesion and a PRO4500 optical engine emitting a 405-nm ultraviolet light (Wintech Digital Systems Technology Corp., Carlsbad, CA) and achieving a nominal 50-µm print resolution. Note that Phantom 14, which contained small channels capable of supporting flow, was fabricated with a higher-resolution pSLA system that used a PRO6500 optical engine (Wintech Digital Systems Technology Corp.) that can achieve a nominal 10-µm print resolution.”

**The short title is the same as the full title, although good alternatives are possible, e.g., “Stereolithography 3D printing of ultrasound phantoms.”**

We would like to thank the reviewer for their suggestion. The short title has now been changed accordingly.

**The number of references may be excessive as many do not directly support the methods in the manuscript.**

References were removed in instances where multiple were provided to (generally) support the same point; this predominately occurred in the Introduction. This resulted in a net reduction of 10 references from the initial submission.

**Line 19. The abstract summarizes the work and its purpose although the key question that was answered is not stated.**

The key point addressed by the work is now explicitly stated at the end of the abstract:

Line 33. “Collectively, these results show that projection-based stereolithography allows for customizable fabrication of complex ultrasound phantoms.”

**Line 48. The insufficiency of current phantoms depends on the QIB, the tissue of interest and the intended application. Elastography and flow are not the only QIBs of interest. For example, quantitative backscatter, attenuation, structure function, form factor, etc., using reference phantom methods are quite robust in liver, muscle and other (admittedly) more homogeneous organs. These methods depend on cellular-level structures, which would require adding scattering material to the 3D printing as described here.**

We agree that the imaging methods given by the reviewer would be very interesting to investigate in future studies. However, given that this work began before the fourth ultrasound-based QIBA initiative, Pulse-Echo Quantitative Ultrasound (PEQUS), was added in 2020, we focused on the original three QIBA initiatives for US-based imaging: elastography and flow imaging (i.e., volume blood flow and intravascular contrast-enhanced imaging). We also agree with the reviewer’s point that methods which rely on cellular-level structures would require the addition of scattering material to the phantoms. To this end, we show in this work that our phantom fabrication method can incorporate silica in suspension, and other work from our group has shown that this platform can be seeded with viable cells, which makes it particularly well-suited for investigating additional biomarkers in the future.

**Line 58. The quote from Ref 10 is referring to precision of QIB measurements specifically in phantoms, which may be overestimated. It is not referring to precision in patients for which many confounders are present but not present in phantoms. How do more complex phantoms contribute to solving this problem?**

The quote from Ref 10 (the entire paragraph is provided below for context) makes the point that the precision calculated in more basic phantoms is often an overestimation of the precision of the same imaging technique *in vivo*. Thus, being able to construct more complex phantoms (e.g., a phantom with tortuous vasculature) would be more likely to present with increased and more realistic variability in imaging results in a “test-retest” scenario where the transducer positioning, contrast injection, etc. are slightly different upon retest. For reasons stated in prior responses, we feel that our phantom fabrication platform can provide some of the “complexity of human targets.”

From ref 10: “*Repeatability studies are typically conducted with all measurements performed at a single clinical site with a specific imaging device. The measurements may be conducted in phantoms, animals, or human subjects. Phantom scans can be repeated several times in sequence, either immediately following one another or separated by a defined time interval. However, phantoms do not represent the complexity of human targets; thus, precision is often overestimated. The ability to perform repeated patient scans is limited because of safety concerns related to radiation exposure for CT or PET procedures, use of contrast media or tracers, or the need to account for kinetic behavior of those agents (eg, a washout period is needed before it is possible to rescan by using contrast media or a tracer). Furthermore, additional patient consent is required for scans that are performed for research purposes and not for clinical care. Human repeatability tests are often limited to two scans performed as test-retest, sometimes called “coffee break” experiments, with only a short break (eg, on the order of minutes or hours) between scans. See Table 4 for a comparison of phantom versus human test-retest study characteristics.”*

**Line 112. “single fabrication step” should probably be changed to “single fabrication session.”**

We would like to thank the reviewer for their suggestion. This phrase has been changed to “single fabrication session.”

**Line 128. “degree necessary” requires more explanation. Perhaps the current limits on printed voxel size and US field of view should be summarized somewhere.**

This sentence has been reworded – and the phrase “degree necessary” has been removed – to improve clarity of the paper:

Line 118. “Ultimately, our method provides researchers with the ability to develop phantoms with customized backscatter and elasticity values as well as with complex vasculature such that they may be made sufficiently realistic and detailed to generally mimic tissue for further development and validation of functional and QIB US imaging techniques.”

**Line 149. What are the x and y dimensions of the stage? Is this the limiting factor for the size of the phantom? All of the phantoms in Fig 2 are very small compared to the fields of view of clinical transducers. In addition, the B-mode images of the phantoms were made at nominally 12-50 MHz, so perhaps it should be stated early in the manuscript that the intended use for the phantoms is for high frequency applications. Can a phantom be fabricated that would contain the entire 3D dimensions of the human transducer? If so, what printing time might be required for Phantom A, if it were made a more useful size of 10 cm L x 10 cm W x 10 cm H? The authors state a printing rate of 3 cm depth per hour (at what x and y dimension?). What is the duration of a complete session needed to fully print a phantom that would encompass the full US transducer 3D field of view?**

The projector field of view is the limiting factor in the X-Y dimension size of the phantoms. We now indicate the maximal print volume possible with the system used in our study:

Line 140. “Briefly, this 3D printing system consisted of a Z-stage coated with Polydimethylsiloxane (PDMS) to avoid PEGDA adhesion and a PRO4500 optical engine emitting a 405-nm ultraviolet light (Wintech Digital Systems Technology Corp., Carlsbad, CA), achieving a nominal 50-µm print resolution, and allowing a maximal print volume (X/Y/Z) of 50x40x65 mm.”

Additionally, we directly address overall print-size limitations with our current setup:

Line 357. “Note that the maximal print size can be increased by increasing the size of the print platform. Additionally, a gelatin block can be cast around finer-detail, printed hydrogels with conventional backfill techniques to increase the effective imaging depth and ensure adequate transducer coupling.”

As was addressed in a previous reply, our phantoms are likely best suited for clinical ultrasound frequencies, in which case print-voxel size becomes significantly smaller than imaging-voxel size. Additionally, the phantom printing time is independent of X-Y dimension size because this entire plane of the phantom is photocured – with the pixel-specific control – at the same time by the projector. Therefore, the only variables that impact printing time are the maximum cure time for each layer, layer thickness, and the number of layers desired. Phantom print time has now been added to Table 1.

**Line 157. Is this discontinuity interface a discrete boundary or is there also a gradient? To the same point, is it possible to create density gradients or only discrete layers?**

Layer thickness, photocuring times, and tartrazine concentration can be modified to change the observed gradients *within* a layer, as well as the magnitude of the discontinuity at each layer boundary. The discontinuity at the interface results from this photocuring gradient within each printed layer, wherein the most photocured (proximal) region of one layer is immediately adjacent (and underneath) to the less photocured (distal) region of the next layer:

Line 347. “US backscatter was generated at the boundary of each printed layer (Fig 3C), resulting from local density mismatches induced at hydrogel-layer boundaries (Fig 3B). In the optical phantoms with FITC-dextran, fluorescence intensity within each layer was inversely correlated with distance from the light source (Fig 3A), indicating that light exposure (i.e., from the projector source) was highest in the most proximal portion of each layer. Because extrusion printing is used, regions with the highest light exposure from one layer are inherently adjacent to regions with the lowest light exposure from the next layer, leading to discontinuities in light exposure through depth (Fig 3A). In B-mode images of matched phantoms, these exposure discontinuities were aligned with the US scattering generated at each layer boundary (Fig 3C), demonstrating that the “stacking” of photocured layers that occurs with extrusion printing produces local density discontinuities that result in hyperechoic regions.”

**Table 1. What was the total printing time to produce each phantom? It would help the reader to understand the advantages you claim for the single-session 3D printing versus other methods.**

We thank the reviewer for the suggestion. We have since added the total phantom print time for each phantom to Table 1.

**Line 197. You reference phantoms 8, 15 and 16. I do not see phantom 16 in this manuscript. What are the different concentrations of silica? How did backscatter coefficient vary with concentration and at what frequencies? How does the presence of the silica affect printing process and needed light exposure time compared to no silica in the formula?**

We apologize for the oversight with the original Phantom 16. This phantom was removed between the initial submission and the resubmission to further focus the paper’s scope. The vestigial reference has been removed, although we have since added a new phantom for experiments suggested by the reviewer, so many of the original phantom numbers have since changed. As noted in Table 1, only one silica concentration was included in this work: 0.1 mg/mL. Although we did not analyze the backscatter coefficient of these phantoms across different frequencies or silica concentrations, this would be an interesting avenue for future work. At the concentration used, silica does not seem to impact the printing process; although, Xanthan gum was added to help the silica particles stay in suspension during the printing process.

**Line 228 and 406. Since these layers are essentially specular reflectors, it may be confusing to refer to backscatter in this context especially since the phantoms have silica scatterers added to the formula. Perhaps you can refer to the effect of exposure time as changing “reflectivity” of acoustic impedance of the layers.**

We have included additional explanation in the manuscript to clarify that the observed backscatter in most cases is specular scattering instead of diffuse scattering. We note that our terminology (i.e., referring to “specular scattering” as “backscattered” sound; e.g., on pg. 227: “…specularly backscattered sound…”) is consistent with that used by Thomas Szabo in his seminal ultrasound textbook, “Diagnostic Ultrasound Imaging: Inside Out.” Because print-voxels are significantly smaller than imaging-voxels for clinical frequencies, it is certainly possible to print diffuse scattering. This was the case with the anisotropic elasticity phantom where adjacent print voxels were exposed to different levels of photocuring, resulting in sub-imaging-voxel variations in acoustic impedance mismatches. We have also clarified that inclusion of a scattering agent, such as silica, would likely be required to generated diffuse scattering resulting in fully developed speckle:

Line 185. “Local US backscatter was generated through voxel-level (acoustic) impedance mismatches at print-layer boundaries. It is important to note that most of the printed backscatter results from specular scattering, as adjacent print voxels were generally photocured to present with the same impedance mismatch. But because adjacent 50-µm print voxels (i.e., below the typical resolution for clinical US) can be made to have varied mismatches (e.g., the anisotropic elasticity phantom), it is possible to generate diffuse scattering. However, the *print* voxels’ relative size and regular periodicity severely limit their ability to generate fully developed speckle, which requires highly dense acoustic scatterers of random phase/amplitude within an *imaging* voxel. Therefore, we fabricated multiple phantoms (Phantoms 7 and 16) with 0.1 mg/mL of 40-μm diameter silica particles (MIN-U-SIL-40, U.S. Silica Co., Mill Creek, OK) to provide tissue-mimicking US speckle, as has been demonstrated in previous work of conventional gelatin-based phantoms [12].”

**The methods for measurement of “backscatter” are not described and the few results are in arbitrary units. Were these measures of pixel “echogenicity” from the B-modes? Ordinarily this incorporates many poorly controlled variables including gain, transmit power, TGC, grey-level LUT, position in FOV, etc. Sound speed estimates were made with the A-line RF signal; was “backscatter” as well?**

We have updated the text with a more detailed explanation of our measurement technique. To summarize the method used, we performed the measurements using pixel echogenicity, while ensuring that gain, transmit power, TGC, dynamic range, and position in the field of view were constant during all imaging sessions:

Line 235. “In addition, because PEGDA degrades via hydrolysis, we assessed the temporal stability of the US signal from the printed targets (i.e., regions with additional cure time) and the base hydrogel (i.e., the region with no additional cure time) over 6 weeks. Matched acquisitions were performed 1, 3, and 6 weeks after the initial time-point to assess imaging temporal stability. All imaging parameters (e.g., gain, transmit power, TGC, dynamic range, and position within the field of view) were constant across all imaging sessions. Signal and contrast-to-noise ratio (CNR) were analyzed from the B-Mode US data across five slices in each printed target for all 4 time-points via the establishment of regions of interest (ROIs) within each printed target and matched ROIs in the base hydrogel region.”

Line 381. “Different cure times in laterally adjacent voxels in the X-Y plane of the phantom produced different echogenicity levels (Fig 3E). Quantifying the backscatter signal in regions of increased exposure in Phantoms 5-7 revealed a nonlinear effect of cure time on US signal, where the first few additional seconds (i.e., the leftmost printed targets in the images) generated a large increase in the US signal relative to the baseline. However, this backscatter signal increased then leveled off after an additional exposure of 5 seconds (i.e., the rightmost printed inclusions; Fig 4C). This increase in US signal within the targets was noted at all three investigated imaging frequencies, and in phantoms with and without Xanthan gum or silica (Phantoms 5-7).”

**Line 333. At 30 MHz, the transit time measurement should be robust while the caliper thickness error for 10 mm might contribute significant uncertainty. What is the accuracy and precision of both measurements in a homogeneous material with known speed of sound and thickness (e.g., nylon, acrylic, etc.)? How many independent measures of thickness and time were made?**

In light of this reviewer comment, speed of sound measurements were redone with a more conventional setup at a frequency range more typical for clinical imaging and with five independent measurements conducted in the sample:

Line 267. “To assess the speed of sound through phantom samples at ultrasonic frequencies, a uniform rectangular phantom with no inclusions (Phantom 8; Fig 2C) was placed on a polished stainless-steel plate and secured with a Saran plastic wrap (S. C. Johnson & Son, Inc., Racine, WI) strip (i.e., the width of the phantom) overtop. The plate was then put in a UMS Research ultrasound measurement water tank containing a 2-axis translation holder (Precision Acoustics Ltd., Dorchester, England); the tank was filled with degassed DI water at a temperature of 20.0 °C. Using a UT320 pulser-receiver (UTEX Scientific Instruments Inc., Mississauga, Ontario, Canada) connected to a DSOX3024A oscilloscope (Keysight Technologies, Santa Rosa, CA), an ultrasound pulse was transmitted and received via an unfocused, circle transducer positioned 25 mm above a region of the plate not containing the phantom. The transducer was then translated laterally to five regions (separated by ~3 mm) overhead the phantom sample, and the pulse-echo scheme was repeated at each. Speed of sound through the sample was then determined using the pulse-echo substitution method [11]. The following unfocused, circle immersion transducers (UTX, Inc., Holmes, NY) were used with these center frequencies (MHz), element diameters (in.), & -6dB bandwidth (%): 2.25, 0.5, & 85; 5.0, 0.5, & 73; 10, 0.375, & 91.”

**Lines 294 and 342. Although you reference your methods as previously published, it would still be valuable to include the important experimental details here, such as frequency.**

We have since updated the text with more experimental details to improve clarity:

Line 289. “Briefly, an acoustic radiation force impulse excitation “push” of 900 cycles at a 6.25-MHz center frequency was focused at a 20-mm depth within the phantom, 5-mm laterally from each target (Fig 2E), to generate a shear wave that was then tracked with pulse-echo US to determine local SWS of the material.”

**Line 390. Mean +/- SD indicates that you made multiple independent measures of each property. How many for each?**

Five independent measures were used for speed of sound analysis, while 10 independent measures were used for shear wave velocity estimation. For values in Table S1 (i.e., characterization of swelling and spatial change over time), duplicated phantoms were fabricated and measured with three independent measurements to provide statistics. Results of US signal and CNR in photocured regions were displayed as the mean and standard deviation values across measurements from five different slices of each contrast target.

**Line 405. Since the sequential printing procedure for each layer produces acoustic interfaces (mis-matches), does this present a problem for phantom design making it difficult to make large homogeneous regions?**

As is shown in Fig 3E, printing presents clearly defined layer boundaries at 50 MHz, but these boundaries become spatially averaged when imaging at lower, clinical-type frequencies, such as 12 MHz, resulting in uniform regions of echogenicity. As is shown in Fig 6A, such a homogenous region (e.g., the “tumor”) can still be printed to then have discrete boundaries relative to other unique features of the phantom (e.g., the phantom background or flanking vessels). This effect of spatial averaging is noted in the manuscript:

Line 374. “Hyperechoic layer boundaries were clearly visible in Phantom 1 (printed with 200-µm layers) when the 50-MHz transducer was used; however, these distinct boundaries were blurred at 30 MHz and difficult to distinguish at all at 12 MHz due to the spatial averaging resulting from lower-frequency imaging (Fig 3D).”

**Line 498. The shear wave speed results show promise for constructing anisotropic phantoms, at least in two dimensions. Researchers in muscle, tendon and nerve with quantitative US will find this has interesting potential for this work.**

We would like to thank the reviewer for this comment. We agree that shear wave anisotropy is an important result from this work.

**Fig 2C. The spherical inclusions are stated to be 5 mm in diameter. What does “0-5 mm” mean here?**

We apologize for the confusion. The symbol to the left of the 5 is not a “0,” but rather it signifies diameter (i.e., diameter of 5 mm). This has been clarified in the figure caption, and the dashes between the diameter symbol and diameter value have been removed to avoid confusion.

**Fig 4 A and B. What is the origin of the dramatic non-uniformities?**

The non-uniformities of the phantom in Fig 4 are likely due to dust mixing with the print mixture as the print stage was open to the environment. This point is now explicitly stated in the text:

Line 399. “Focal regions of hyperechogencity (e.g., X,Y = 3,2 in Fig. 4A) are likely a result of dust contamination during printing, an effect that can be mitigated by physically shielding the open print stage from the environment.”

**Ref 44 is incomplete and it is not clear what type of publication this is (patent, book, etc.).**

We would like to thank the reviewer for pointing out this issue. The reference in question was for a white paper published by SuperSonic Imagine; however, we have since removed the reference entirely in response to a previous reviewer comment.

**Ref 52 and 53 appear to be (almost) duplicates, referring to the same publication.**

We apologize for the duplication, which has since been corrected.

***Reviewer #2:***

**The authors present interesting developments in the realization and characterization of 3D printed US phantoms. Several samples and designs were fabricated and analyzed, while varying manufacturing parameters. Overall a very nice work that should be of interest to our readers.**

We would like to thank the reviewer for their comments in support of our work.

**One question, that perhaps this reviewer missed, is did the authors measure the as-printed feature dimensions in comparison to their original CAD model? Some understanding of the geometric fidelity of this process, if available, might also add value for the generation of complex 3D phantom structures.**

While this was not included in the original submission, we have performed additional experiments to add this information to the manuscript. Thus, the precision of measurements of a given geometric feature (i.e., lumen that extends across the phantom) within a phantom, the repeatability of geometric features for matched phantoms, the accuracy of geometric features compared to CAD dimensions, and the geometric stability of phantoms over 31 days after printing were all assessed in two formulations (i.e., with and without the addition of GelMA) and at multiple storage conditions (DI water vs PBS at 4⁰C or 25⁰C):

Line 251. “To assess print stability and fidelity, rectangular phantoms with a 1mm-inner-diameter linear lumen (Phantom 9, 10; Fig 2D) were printed and immediately stored in DI water or PBS at 4° or 25°C. In addition to a standard PEGDA formulation (Phantom 9), a second PEGDA formulation containing GelMA (Phantom 10) was also tested. B-mode volumetric imaging (30 MHz; MS400) was performed on matched pairs of each formulation stored at each temperature, with the phantom lumen positioned perpendicular to the transducer axis for four time-points: 0 (i.e., immediately after printing), 1, 2, & 4 weeks following printing. For each time-point, three imaging slices separated by 3-4 mm were chosen, and the midsection extent in the Y- and Z-axis of the rectangular phantom’s walls and lumen was measured using digital calipers in the VevoLAB analysis package (FUJIFILM VisualSonics, Toronto, ON, Canada). Precision was assessed by the standard deviation of measurements for a constant geometric feature (e.g., lumen) across three locations for the same phantom; reproducibility was assessed by comparing the percent difference in geometric features between two matched phantoms; stability was assessed by tracking the change in geometric features for a given phantom over time; and accuracy (i.e., print fidelity) was assessed by comparing the dimensions of printed GelMA-containing phantoms to their CAD dimensions.”

Line 403. “Hydrogels containing GelMA (Phantom 10) stored in PBS were morphologically most stable (Table S1), experiencing an average (i.e., for both dimensions of the phantom body and lumen) absolute dimensional change compared to their CAD design of 5.2% (day of print) and 4.8% (31 days after print) at 4°C and 4.4% (day of print; this value was also used as the estimate for accuracy) and 6.6% (31 days after) at 25°C. Hydrogels without GelMA (Phantom 9) were less stable, experiencing an average absolute dimensional change compared to their CAD design of 12.4% (day of print) and 26.1% (31 days after print) at 4°C and 8.2% (day of print) and 21.1% (31 days after print) at 25⁰C. Hydrogels stored in DI water, regardless of storage temperature, immediately experienced significant (>20%) dimensional increases (i.e., both lumen and body) when compared to their initial CAD dimensions. Across all combinations, reproducibility measurements between matched phantoms differed by an average of 2.8%, while the average precision between measurements within the same phantom was 3.0%. Although print accuracy was estimated at 4.4%, such a metric can be difficult to characterize as interaction of the phantom with the measurement environment (e.g., PBS) can quickly cause changes from the original print geometry. In future work, we intend to investigate different PEG crosslinking chemistries that are more resistant to hydrolysis.”
