# Supplementary material for: Projection-based stereolithography for direct 3D printing of heterogeneous ultrasound phantoms
Source: PLoS One. 2021 Dec 9;16(12):e0260737. doi: 10.1371/journal.pone.0260737 (PMC8659365; doi:10.1371/journal.pone.0260737)
Supplement: Attachment — Submitted filename: ReveiwerReplyFinal.docx [file pone.0260737.s010.docx]

*We would like to thank the editor for arranging the review of our manuscript and the referees for their helpful comments. We have addressed all reviewer comments and suggestions and feel that the quality and clarity of our manuscript has improved as a result. Reviewer comments are provided verbatim in bold text, and our reply to each comment is given immediately following with excerpts from the revised manuscript provided in quotations; changes to the manuscript are denoted with yellow highlighting.*

**Reviewer Comments**

**1. This kind of phantom was mainly used in ultrasound imaging study, therefore, the ultrasound properties of the phantom, such as speed of sound and attenuation, should be presented.**

We would like to thank the reviewer for their comment. We certainly agree that US-related properties of the phantom should be well characterized. Thus, in our submission we characterize the speed of sound, relationship between photocuring and relative US backscatter, temporal stability of relative US backscatter, and elasticity of phantom samples, with the following summary of these results provided in the manuscript.

Speed of sound:

“We also characterized the group velocity through our hydrogel formulation (Phantom 9) to be 1527±1, 1523±2, & 1527±1 m/s for 2.25, 5, & 10MHz, respectively.”

Backscatter as a function of photocuring time:

“Quantifying the backscatter signal in regions of increased exposure in Phantoms 5-7 revealed a nonlinear effect of cure time on US signal, where the first few additional seconds (i.e., the leftmost printed targets in the images) generated a large increase in the US signal relative to the baseline. However, this backscatter signal increased then leveled off after an additional exposure of 5 seconds (i.e., the rightmost printed inclusions; Fig 4C). This increase in US signal within the targets was noted at all three investigated imaging frequencies, and in phantoms with and without Xanthan gum or silica (Phantoms 5-7).”

Backscatter as a function of time:

Because PEGDA hydrogels can degrade over time via hydrolysis [44], we tested the temporal stability of the backscatter signal they generate. The US signal from all 1.5-mm printed targets in Phantom 5 (Fig 4A) decreased over time (Fig 4C). However, the CNR remained relatively stable over 6 weeks of storage and imaging (Fig 4D).

Elasticity:

“Shear wave imaging data for the compliant elasticity phantom (Phantom 12) showed mean shear wave velocities between 1.6±0.1 and 2.3±0.1 m/s within regions of 5 and 12.5 seconds of cure time, respectively (S2 Table). These shear wave velocities correspond to Young’s moduli of 7.6 and 15.6 kPa, respectively, assuming a linear, isotropic, elastic medium; these values are within the range reported for *in vivo* imaging of soft tissue [41]. The stiff elasticity phantom (Phantom 13) presented a similar trend of increasing shear wave velocity with cure time; however, Young’s modulus estimates within the inclusions were significantly higher than typical soft tissue values, ranging from 72.9 to 123.6 kPa (S2 Table).”

Unfortunately, our experimental setup was not configured to conduct accurate attenuation measurements on the phantom samples. However, in previous work done by Aliabouzar *et al.* on similar PEGDA-based samples, in addition to measuring comparable sound speeds to what we report, they also measured the US attenuation of these samples. Thus, we have included their attenuation findings in the Discussion as reference:

“This result is similar to work done by Aliabouzar *et al.*, which reported sound speed values between 1500-1600 m/s for the same class of hydrogel [34]. This prior study also demonstrated that similar 3D-printed PEGDA-based samples as those investigated in our study present with US attenuation that is consistent with soft tissue, reporting average attenuation values of 0.54, 0.85, & 1.27 dB cm^-1^ MHz^-1^ when measured with center frequencies of 2.25, 5, 10 MHz, respectively.”

**2. The 2D imaging of Bmode, color Doppler, shear wave elastography should be provided in this study.**

We thank the reviewer for this suggestion. We agree that inclusion of 2D images of these modalities strengthens the manuscript. While 2D B-mode images were already included in Figures 3, 5, and S2, we have added representative images of shear-wave propagation as new Supplemental Figure 3 (S3 Fig) and added representative 2D Doppler images overlaid 2D B-mode images as new Supplemental Figure 4 (S4 Fig) and. Representative 2D B-mode images of phantoms with varying silica concentrations have also been included as new Supplemental Figure 5 (S5 Fig).

**3. Typically, scatterer is important for ultrasound imaging, however, uniform scatterer distribution can not be seen in the figure 3 and figure 4, it should be discussed. In addition, some strong reflection like bubbles can been seen in figure 4, the author should also discuss it and explain how to avoid the the bubbles in phantom cooking.**

In last the submission, Table 1 mistakenly showed Phantom 7 (i.e., which had silica) as included in Figure 3. However, the phantoms depicted in this figure do not include silica, and thus the resulting backscatter is entirely due to the print-layer discontinuity described in the manuscript. We apologize for this oversight; Table 1 has since been updated to correct this error.

Regarding the scattering seen in the phantom in Figure 4B, which did contain silica scatterers (Phantom 7; 0.1 mg/mL), we agree that the presented backscatter is not as uniform and dense as typically observed from soft tissue. Thus, we have now included an additional phantom with increased silica concentration (i.e., Phantom 8; 1 mg/mL of silica) that provides scattering more consistent with soft-tissue speckle. We have also provided 2D B-mode comparisons of this new phantom (Phantom 8), the old lower-concentration silica phantom (Phantom 7), and a silica-free phantom (Phantom 6) in a supplemental figure (S5 Fig). As part of future work, silica and xanthan gum (which mitigates silica-particle settling) concentrations should be optimized in combination with print-layer thickness (i.e., to ensure complete photocuring in the presence of additional scatterers) to ensure that minimal local silica settling/aggregation occurs during photocuring of phantom layers. This point has now been made in the revised manuscript:

“Phantoms with silica concentrations of 1 mg/mL (i.e., Phantom 8) provide scattering more consistent with speckle than phantoms with lower silica concentrations (i.e., 0.1 mg/mL; Phantom 7) or phantoms with no silica added (i.e., Phantom 6; S5 Fig). As part of future work, silica and xanthan gum concentrations can be optimized in combination with print-layer thickness (i.e., to ensure complete photocuring in the presence of additional scatterers) to ensure that silica particles do not settle and/or aggregate during the phantom photocuring process.”

Finally, we agree with the reviewer that some strong reflections are seen in Figure 4, which are most obvious in the phantom lacking silica additives (i.e., Phantom 5 in Fig 4A). These focal regions of hyperechogenicity (e.g., X,Y = 3,2 in Fig 4A) are likely a result of dust contamination during printing. In the future, this artifact can be mitigated by physically shielding the open print stage from the environment and/or by placing the stage in a (slight) negative-pressure vacuum hood. We now discuss this issue and potential remedies in the manuscript:

“Focal regions of hyperechogenicity (e.g., X,Y = 3,2 in Fig 4A) are likely a result of dust contamination during printing, an effect that can be mitigated by physically shielding the open print stage from the environment and/or by placing the stage in a (slight) negative pressure vacuum hood.”

**4. Is this phantom water based? Should the phantom be used and stored in water? Did the author consider any preservative in this study?**

We would like to thank the reviewer for pointing out an important aspect of the phantoms’ composition that needed clarification. Yes, the phantoms are water-based as the PEGDA and GelMA macromers are dissolved in PBS as part of fabrication. This has now been explicitly stated in the manuscript:

“PEGDA and GelMA macromers were dissolved in phosphate-buffered saline (PBS) to make pre-polymer solutions.”

Phantoms were imaged using either DI water, PBS, or ultrasound gel for coupling, and we assessed phantom stability when stored in either DI water or PBS. S1 Table details the effects of DI-water or PBS storage conditions at 4°C or 25°C. As is now stated, PBS provides a better storage condition compared to DI water because of the osmotic imbalance between the PBS-based phantoms and (salt-free) DI water. A summary of these results is included the paper:

“Hydrogels containing GelMA (Phantom 11) stored in PBS were morphologically most stable (S1 Table), experiencing an average (i.e., for both dimensions of the phantom body and lumen) absolute dimensional change compared to their CAD design of 5.2% (day of print) and 4.8% (31 days after print) at 4°C and 4.4% (day of print; this value was also used as the estimate for accuracy) and 6.6% (31 days after) at 25°C. Hydrogels without GelMA (Phantom 10) stored in PBS were less stable, experiencing an average absolute dimensional change compared to their CAD design of 12.4% (day of print) and 26.1% (31 days after print) at 4°C and 8.2% (day of print) and 21.1% (31 days after print) at 25⁰C. Hydrogels stored in DI water, regardless of storage temperature, immediately experienced significant (>20%) dimensional increases (i.e., both lumen and body) when compared to their initial CAD dimensions, an expected result given the osmotic imbalance between the PBS-based phantoms and (salt-free) DI water.”

Finally, while the inclusion of preservatives would be an interesting avenue to investigate in the future, we have not yet explored their use as such preservatives could potentially interfere with the platform’s demonstrated viability for embedded living cells:

“The platform’s demonstrated viability for embedded living cells [33] also offers unique opportunities for highly precise and potentially more realistic *in vitro* imaging studies.”
